# Partial-volume correction in dynamic PET-CT: effect on tumor kinetic parameter estimation and validation of simplified metrics

**DOI:** 10.1186/s13550-019-0483-z

**Published:** 2019-02-04

**Authors:** M. C. F. Cysouw, S. V. S. Golla, V. Frings, E. F. Smit, O. S. Hoekstra, G. M. Kramer, R. Boellaard, on behalf of the QuIC-ConCePT Consortium

**Affiliations:** 1 Department of Radiology and Nuclear Medicine, Amsterdam UMC, Vrije Universiteit Amsterdam, Cancer Center Amsterdam, De Boelelaan 1117, Amsterdam, the Netherlands; 2 Department of Thoracic Oncology, Netherlands Cancer Institute, Plesmanlaan 121, Amsterdam, the Netherlands

**Keywords:** Dynamic PET-CT, Kinetic parameter estimation, Partial-volume correction, Oncology

## Abstract

**Background:**

Partial-volume effects generally result in an underestimation of tumor tracer uptake on PET-CT for small lesions, necessitating partial-volume correction (PVC) for accurate quantification. However, investigation of PVC in dynamic oncological PET studies to date is scarce. The aim of this study was to investigate PVC’s impact on tumor kinetic parameter estimation from dynamic PET-CT acquisitions and subsequent validation of simplified semi-quantitative metrics. Ten patients with EGFR-mutated non-small cell lung cancer underwent dynamic ^18^F-fluorothymidine PET-CT before, 7 days after, and 28 days after commencing treatment with a tyrosine kinase inhibitor. Parametric PVC was applied using iterative deconvolution without and with highly constrained backprojection (HYPR) denoising, respectively. Using an image-derived input function with venous parent plasma calibration, we estimated full kinetic parameters *V*_T_, *K*_1_, and *k*_3_/*k*_4_ (BP_ND_) using a reversible two-tissue compartment model, and simplified metrics (SUV and tumor-to-blood ratio) at 50–60 min post-injection.

**Results:**

PVC had a non-linear effect on measured activity concentrations per timeframe. PVC significantly changed each kinetic parameter, with a median increase in V_T_ of 11.8% (up to 25.1%) and 10.8% (up to 21.7%) without and with HYPR, respectively. Relative changes in kinetic parameter estimates vs. simplified metrics after applying PVC were poorly correlated (correlations 0.36–0.62; *p* < 0.01). PVC increased correlations between simplified metrics and V_T_ from 0.82 and 0.81 (*p* < 0.01) to 0.90 and 0.88 (*p* < 0.01) for SUV and TBR, respectively, albeit non-significantly. PVC also increased correlations between treatment-induced changes in simplified metrics vs. V_T_ at 7 (SUV) and 28 (SUV and TBR) days after treatment start non-significantly. Delineation on partial-volume corrected PET images resulted in a median decrease in metabolic tumor volume of 14.3% (IQR − 22.1 to − 7.5%), and increased the effect of PVC on kinetic parameter estimates.

**Conclusion:**

PVC has a significant impact on tumor kinetic parameter estimation from dynamic PET-CT data, which differs from its effect on simplified metrics. However, it affected validation of these simplified metrics both as single measurements and as biomarkers of treatment response only to a small extent. Future dynamic PET studies should preferably incorporate PVC.

**Trial registration:**

Dutch Trial Register, NTR3557.

**Electronic supplementary material:**

The online version of this article (10.1186/s13550-019-0483-z) contains supplementary material, which is available to authorized users.

## Background

In clinical oncology, positron-emission tomography (PET) is a valuable tool allowing guidance of treatment on a per-patient basis [1]. Clinical decision-making using PET-CT is commonly limited to visual analysis, where local disease and the presence of nodal or distant metastases is evaluated [2, 3]. However, since PET is an inherently quantitative technique, it may also be used for quantitative assessment of tumor metabolic, proliferative, or drug targeting characteristics [1, 4, 5].

For quantitative PET-CT to be of practical clinical utility, metrics need to be easily extracted from static whole-body PET-CT images as performed in routine clinical practice. To this end, standardized uptake values (SUV) are typically used as simplified semi-quantitative measures of tracer uptake [6]. However, pharmacokinetic modeling using dynamic PET-CT acquisitions with arterial or venous blood sampling is an essential first step to technically validate the clinical use of these simplified metrics as biomarkers of, e.g., response to treatment [4, 5, 7, 8].

As is well known, quantification of tracer distribution on PET-CT scans is hampered by several sources of error. Among these are attenuation, Compton scatter, random coincidences, and decay, all accounted for by contemporary image reconstruction algorithms. However, due to the inherently limited spatial resolution of PET-CT, acquired images still suffer from partial-volume effects [9]. Partial-volume effects lead to spill-in and spill-out of measured activity distributions, generally resulting in net underestimations of tracer uptake, the extent of which depend on tumor size, shape, and contrast [9]. Hence, partial-volume correction (PVC) is needed for accurate quantification, especially for small and/or heterogeneous lesions [9–12].

In oncological studies, PVC has been predominantly applied to static PET-CT images (in contrast with brain [13–22] or cardiac [23, 24] PET imaging). However, in dynamic acquisitions, the activity spill-over in and from tumors due to partial-volume effects may vary over time. The impact of PVC on tumor kinetic parameter estimates could therefore differ from its impact on simplified measures of uptake. Consequently, it may not only affect absolute quantitative reads but also validation of simplified parameters for clinical implementation.

The present study aims to evaluate the impact of frame-wise parametric PVC in dynamic PET-CT studies on tumor kinetic micro- and macroparameter estimations, and evaluate the correlation between its effect on kinetic parameters and simplified metrics. Secondly, PVC’s effect on technical validation of simplified ^18^F-fluorothymidine (^18^F-FLT) PET-CT metrics as biomarkers of response to treatment of non-small cell lung cancer (NSCLC) with tyrosine kinase inhibitors (TKI) will be investigated.

## Methods and materials

### Patients

The present study is a retrospective analysis of a prospective cohort study [5]. Patients with metastatic epidermal growth factor receptor (EGFR) mutated NSCLC scheduled for treatment with an EGFR-TKI were included. All patients were scanned with ^18^F-FLT PET-CT on three occasions: at baseline, 7 days after, and 28 days after commencing treatment with a TKI (gefitinib or erlotinib), respectively. The Amsterdam UMC (location VUmc) institutional review board approved this study (Dutch Trial Register, NTR3557), and all included patients provided informed consent for study participation.

### PET-CT image acquisition and reconstruction

The EARL-compliant imaging protocol was described previously [5]. All scans were acquired on a Philips Gemini TF-64 PET-CT scanner (Philips Healthcare). Patients were instructed not to eat 4 h prior to each scan. A thoracic field of view was placed such that it contained the primary tumor, using a transmission scan for positioning. A 60-min dynamic PET acquisition started directly after injection of 370 MBq ^18^F-FLT in 5 mL saline (flushed with 20 mL saline). Afterwards, a low-dose CT was acquired for attenuation correction (120 kV, 50 mAs). The PET emission scan was binned into 36 frames with varying durations (1 × 10, 8 × 5, 4 × 10, 3 × 20, 5 × 30, 5 × 60, 4 × 150, 4 × 300, and 2 × 600 s). Images were reconstructed with a time-of-flight 3D row action maximum likelihood algorithm (3 iterations, 33 subsets), as provided by the vendor, with corrections for Compton scatter, random coincidences, attenuation, and decay. PET image dimensions were 144 × 144 × 45 voxels with voxel dimensions of 4 × 4 × 4 mm. Venous blood samples were drawn at 5, 10, 20, 30, 40, and 60 min post-injection of ^18^F-FLT. From each sample, the whole blood and plasma activity concentrations and parent fractions were measured.

### Image processing

For PVC, we applied a post-reconstruction iterative deconvolution algorithm (Lucy-Richardson [LR]) [25]. This parametric (voxel-wise) method aims to deblur images by iteratively correcting the activity spill-over, only assuming approximate knowledge of the PET-CT scanner’s spatial resolution. We set the full-width at half-maximum (FWHM) of a spatially invariant Gaussian point spread function at 7.5 mm, as previously calibrated in phantom experiment for the used scanner [11], with ten iterations allowing for sufficient convergence. PVC was applied to each image frame. As iterative deconvolution is known to result in lower signal-to-noise ratios (SNR), in order to evaluate effect of image noise we additionally applied a highly constrained backprojection (HYPR) algorithm shown to improve SNR for dynamic PET studies [26, 27]. Iterative deconvolution was applied without and with HYPR denoising (denotated as LR and LR + HYPR, respectively). HYPR settings were optimized, comparing a single composite image (HYPR_single_) and several moving frame composite images (HYPR_moving_), using a Gaussian 7.5 mm FWHM low-pass filter (F). The HYPR implementation can be described as follows [21, 26]:

1 $$ {I}_H={I}_c\times {I}_w $$

2 $$ {I}_c=\sum {I}_i\times {\Delta  t}_i $$

3 $$ {I}_w=\frac{F\otimes {I}_o}{F\otimes {I}_c} $$

where *I*_*H*_ is the HYPR image; *I*_*c*_ is the composite image, which is a duration weighted summed average of either all frames in the dynamic image (HYPR_single_) or a set of frames around the to be denoised frame (HYPR_moving_), with ∆*t*_*i*_ as the individual frame duration; *I*_*o*_ is the original dynamic frame being denoised; and *I*_*w*_ is the weighting image computed as the ratio between the spatially filtered original frame and spatially filtered composite image.

### Kinetic modeling and semi-quantitative analysis

Lesions were delineated using in-house developed software (VU University Medical Center) on a volume-of-interest (VOI) basis [28]. Tumor delineation was performed on a summation of the last three PET frames of the original (non-PVC) image. In short, a rough manual delineation was performed, warranting all peak ^18^F-FLT-avid tumor activity was contained in the VOI and no non-tumor structures with high uptake were included. Second, this VOI was shrunk to an isocontour based on 50% of the peak value (mean activity in a 12-mm sphere positioned to provide the highest uptake value), with correction for local background activity. VOIs were then projected onto each frame of both the original and partial-volume corrected PET images to acquire time activity curves from both the datasets (without and with PVC). To explore the effect of PVC on tumor delineation, tumors were also delineated on the LR + HYPR images using the same approach. Metabolically active tumor volume (MATV) was defined as the sum of voxel volumes within a VOI.

A 2 × 2 voxel (8 × 8 mm) region was placed centrally in ascending aorta on five adjacent slices to acquire an image-derived input function (IDIF), aiming to avoid partial-volume effects. Parent plasma input functions were generated by calibrating IDIFs using the activity concentrations measured in the venous blood samples, and correcting for metabolites and plasma-to-blood ratio. Full quantitative parameters derived from kinetic modeling and simplified measures were extracted using in-house developed software in MATLAB. We used a reversible two-tissue model with blood volume parameter, which has been identified as the optimal compartment model for ^18^F-FLT by Frings et al. [5]. Pharmacokinetic parameters rate of influx of the tracer from blood to tissue (*K*_1_), volume of distribution (*V*_T_), and binding potential (BP_ND_) of each lesion were derived using non-linear regression, where:

4 $$ {V}_T=\frac{K1}{k2}\left(1+\frac{k3}{k4}\right) $$

5 $$ BP=\frac{k3}{k4} $$

*V*_T_ served as the preferred reference parameter for validation of simplified metrics for ^18^F-FLT [5]. The simplified metrics, mean SUV, and tumor-to-blood ratio (TBR; parent plasma) were derived at a 50–60 min post-injection scan interval, where:

6 $$ \mathrm{SUV}=\frac{\mathrm{activity}\ \mathrm{concentration}\ \left[\frac{\mathrm{Bq}}{\mathrm{mL}}\right]}{\left(\frac{\mathrm{injected}\ \mathrm{activity}\left[\mathrm{Bq}\right]}{\mathrm{lean}\ \mathrm{body}\ \mathrm{mass}}\right)} $$

7 $$ \mathrm{TBR}=\frac{\mathrm{tumor}\ \mathrm{activity}\ \mathrm{concentration}\ \left[\frac{\mathrm{Bq}}{\mathrm{mL}}\right]}{\mathrm{blood}\ \mathrm{activity}\ \mathrm{concentration}\ \left[\frac{\mathrm{Bq}}{\mathrm{mL}}\right]} $$

### Statistical analysis

Data were described as mean with standard deviation (SD), median with interquartile range (IQR), minimum and maximum. Correlations between pairwise data were investigated using Spearman correlation. To assess technical validation of simplified metrics, we assessed correlations between both single measurements of kinetic parameter estimations and simplified metrics as well as correlations between relative changes in these parameters during treatment. Differences were tested using the Wilcoxon signed rank test (two related) or the Friedman test (multiple related), with significance level *p* < 0.05. SPSS Statistics v22 (IBM) was used for statistical analyses.

## Results

### Patients

Ten patients with EGFR-mutated NSCLC were included, consisting of four men and six women with a mean age of 64 ± 8 years. Treatment consisted of gefitinib and erlotinib in seven and three patients, respectively. In one patient, the baseline scan was not evaluable due to scanner failure (scan at 7 and 28 days could still be used for lesion-based analyses). Another patient had no visible lesions at PET-CT. Twenty-four suspected lesions were detected on ^18^F-FLT PET-CT [5].

### HYPR optimization

**Fig. 1:**
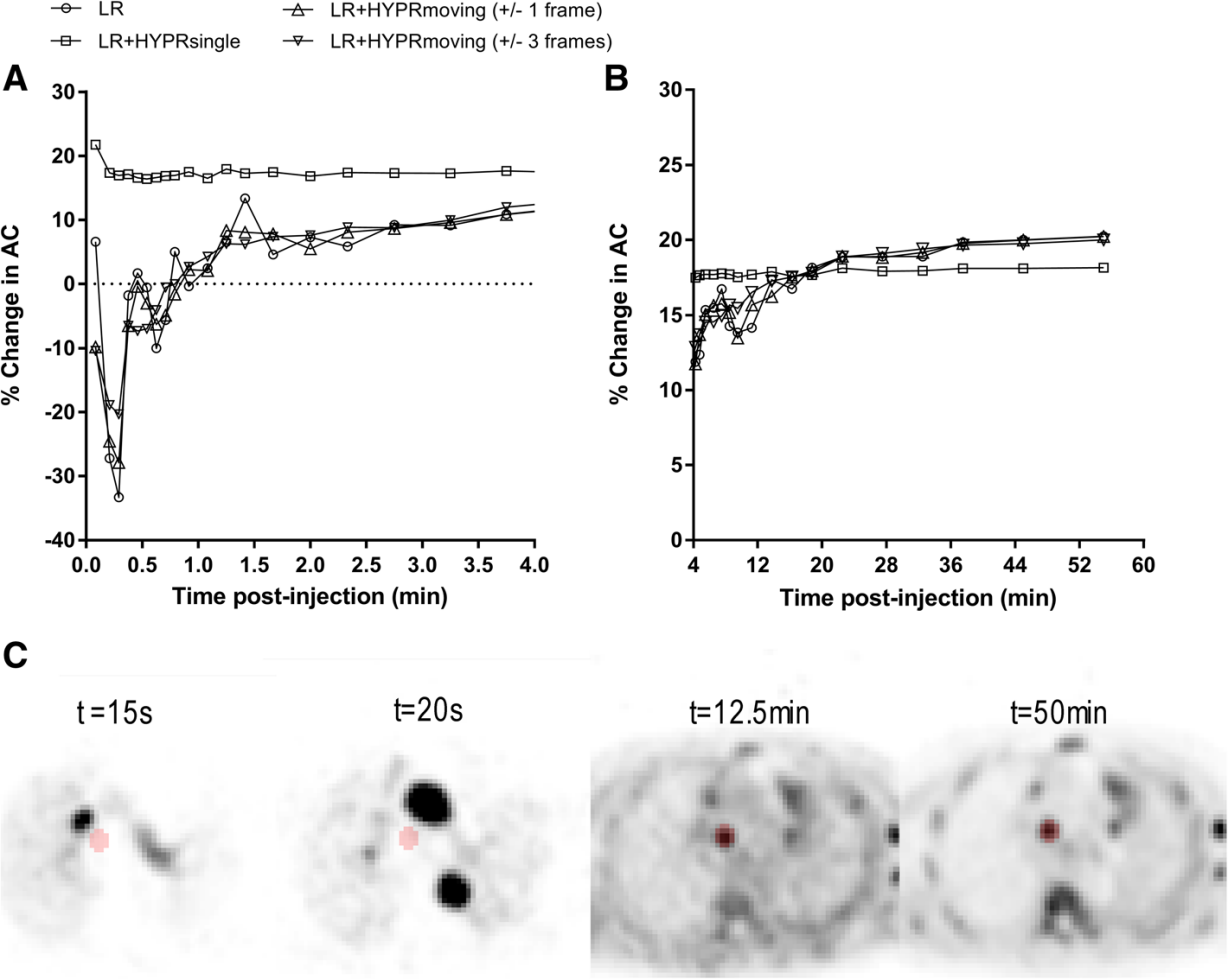
Time-activity curves of relative change in activity concentrations (AC) after PVC using several HYPR settings. Frames of 0–4 min (**a**) and 4–60 min (**b**) post-injection. Results of a typical mediastinal lymph node metastasis are shown. Note the temporality of PVE with a spill in at early timeframes. Corresponding original PET images (**c**) with the lesion volume-of-interest in red demonstrate blood pool activity near the VOI and increasing tumor-to-background contrast over time

A single composite (HYPR_single_) provided most SNR improvement (Additional file 1: Figure S1). However, it eliminated the temporal dynamics of PVC (Fig. 1). A HYPR_moving_ setting with a composite image consisting of ± 3 frames relative to the denoised frame provided an adequate trade-off between SNR improvement and partial-volume correction and was hence used in further analyses.

### Image-derived input functions

We verified the assumption that partial-volume effects do not affect ascending aorta-derived IDIFs (based on the 2 × 2 voxel VOI approach used that minimized or avoided partial volume effects). First, PVC introduced only small relative differences in IDIF area under the curve (AUC; Table 1), which were mitigated by HYPR_moving_ and reduced to 0% by HYPR_single_ (the latter providing most noise mitigation). As a consequence, IDIF AUCs of uncorrected and PVC images were highly correlated (Additional file 1: Table S1). Similar results were observed for parent plasma calibrated input curves. Also, kinetic parameter estimates derived from uncorrected images using uncorrected vs. PVC input functions were very similar (Additional file 1: Table S2); small but significant differences in *V*_T_ and *K*_1_ were observed for LR and LR + HYPR_moving_ IDIFs, but not when HYPR_single_ was applied. Therefore, we continued our analyses using the parent plasma calibrated input functions derived from uncorrected PET images.

**Table 1:** Median relative differences (% with IQR) in IDIF AUC of PVC-images compared to uncorrected images

	Entire curve		Peak only (2.5 min)	
	Image-derived	PP calibrated	Image-derived	PP calibrated
LR	−0.8 (−1.2 to 0.6)	−0.7 (− 1.3 to − 0.2)*	− 2.0 (− 3.4 to − 0.9)*	− 1.8 (− 3.7 to − 0.9)*
LR + HYPR_moving_	− 0.7 (− 1.2 to 0.6)	−0.6 (− 1.1 to − 0.1)*	− 2.2 (− 3.2 to − 0.5)*	− 2.0 (− 3.3 to − 1.1)*
LR + HYPR_single_	− 0.8 (− 1.2 to 0.6)	0.0 (0.0 to 0.0)	−0.9 (− 1.2 to 0.7)	0.0 (− 0.1 to 0.1)

*p < 0.05. *PP* parent plasma

### Kinetic parameter estimates and simplified metrics

Relative differences between uncorrected and PVC data for *K*_1_, *V*_T_, BP_ND_, SUV, and TBR are presented in Table 2. Both LR and LR + HYPR_moving_ significantly (*p* < 0.001) increased each parameter. Overall, LR provided larger changes in parameters than LR + HYPR_moving_ for both kinetic parameters and simplified metrics. Regarding kinetic parameters, largest changes were seen for *V*_T_, which was increased by median 13.2% up to 25.1% using LR. Changes in *K*_1_ and BP_ND_ were very similar (median 6.8% and 6.0%, respectively, using LR). Changes in SUV and TBR after PVC were almost identical, as expected, and were comparable to changes in *V*_T_. LR and LR + HYPR_moving_ decreased *V*_T_, *K*_1_, and BP_ND_ in some lesions, but only provided increases for SUV and TBR. Changes in *V*_T_, *K*_1_, and BP_ND_ after PVC had low but significant correlations with changes in SUV and TBR after PVC (Table 3); highest correlations were seen between relative changes in *V*_T_ and changes in SUV and TBR (up to 0.62).

**Table 2:** Relative changes (%) in kinetic parameter estimates and simplified metrics after PVC

	Mean	Median	SD	IQR	Min	Max	*p* value
LR							
*V*_T_	11.8	13.2	7.1	6.0–16.4	− 15.2	25.1	< 0.001
*K*_1_	6.6	6.8	7.5	2.6–11.1	− 16.7	32.3	< 0.001
BP	6.1	6.0	8.8	2.1–10.7	− 21.9	34.6	< 0.001
SUV	13.1	13.2	6.1	7.3–17.1	3.3	28.4	< 0.001
TBR	13.1	13.2	6.1	7.3–17.1	3.3	28.3	< 0.001
LR + HYPR							
*V*_T_	10.8	11.7	6.1	6.1–15.5	− 13.6	21.7	< 0.001
*K*_1_	5.7	4.3	6.9	2.3–10.0	− 14.9	25.1	< 0.001
BP	3.7	4.4	6.4	0.1–7.1	− 20.6	19.8	< 0.001
SUV	12.6	12.9	5.8	7.0–16.7	2.1	24.7	< 0.001
TBR	12.8	12.9	6.0	7.0–17.0	3.1	27.3	< 0.001

**Table 3:** Correlation (Spearman, with 95% confidence intervals) between PVC-induced relative changes in kinetic parameter estimates and simplified metrics

	*V*_T_	*K*_1_	BP
LR			
SUV	0.58* (0.38–0.73)	0.61* (0.42–0.75)	0.51* (0.30–0.68)
TBR	0.58* (0.38–0.73)	0.61* (0.42–0.75)	0.51* (0.30–0.68)
LR + HYPR			
SUV	0.62* (0.43–0.75)	0.47* (0.24–0.65)	0.36* (0.11–0.56)
TBR	0.62* (0.43–0.75)	0.48* (0.26–0.66)	0.36* (0.12–0.57)

*p < 0.01

**Fig. 2:**
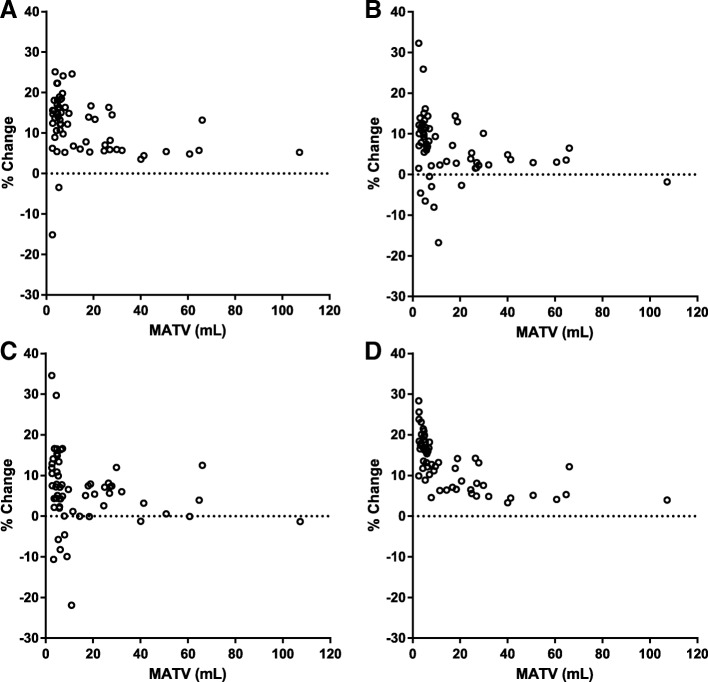
Relative change (%) in quantitative parameters after PVC (LR) as a function of lesion MATV (mL) for *V*_T_ (**a**), *K*_1_ (**b**), BP (**c**), and SUV (**d**). TBR is not displayed since it was virtually identical to SUV

We plotted relative changes in *V*_T_, *K*_1_, BP_ND_, and SUV after PVC as a function of lesion (original) MATV to provide insight into the effect of lesion size on PVC performance (Fig. 2). For LR, the correlations between MATV and relative change in *V*_T_, *K*_1_, BP_ND_, SUV, and TBR were − 0.39, − 0.47, − 0.36, − 0.80, and − 0.80, respectively (*p* < 0.01). For LR + HYPR, these correlations were − 0.43, − 0.34, − 0.24, − 0.81, and − 0.80, respectively (*p* < 0.01, except for BP_ND_; *p* = 0.07).

**Fig. 3:**
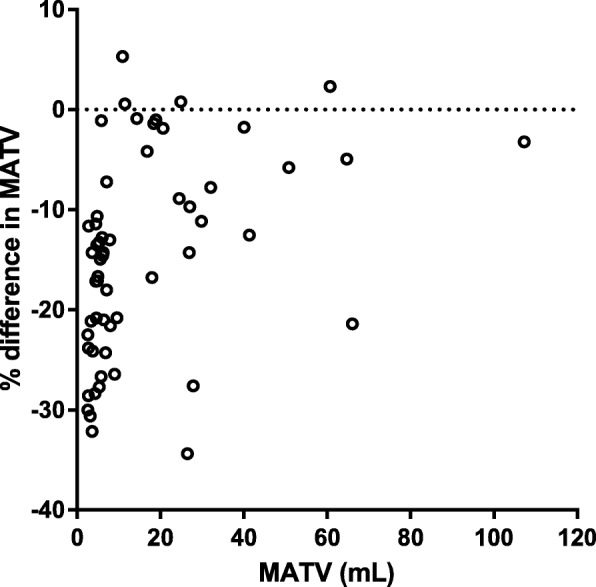
Relative difference (%) in lesion MATV (mL) between uncorrected and PVC images (LR + HYPR) as function of MATV on uncorrected images. *Y*-axis was scaled to − 40%; for one lesion of 5.8 mL MATV was 69% smaller on PVC image

Compared to tumor delineation on uncorrected images, delineation on partial-volume corrected images (LR + HYPR_moving_) provided a median relative decrease in MATV of 14.3%, (IQR − 22.1 to − 7.5, minimum − 69.2, maximum 5.3; Fig. 3). Also, the effect of PVC on kinetic parameters and simplified metrics was higher when using VOIs generated on PVC images compared to when using original VOIs (Additional file 1: Table S3). Here, largest increases after PVC were seen for *V*_T_, SUV, and TBR with median increases of 13.9% (IQR 7.6–18.7; max 37.8%), 15.8% (IQR 8.4–20.4; max 31.5), and 15.8% (IQR 8.4–20.7; max 34%), respectively.

### Technical validation of simplified metrics

**Fig. 4:**
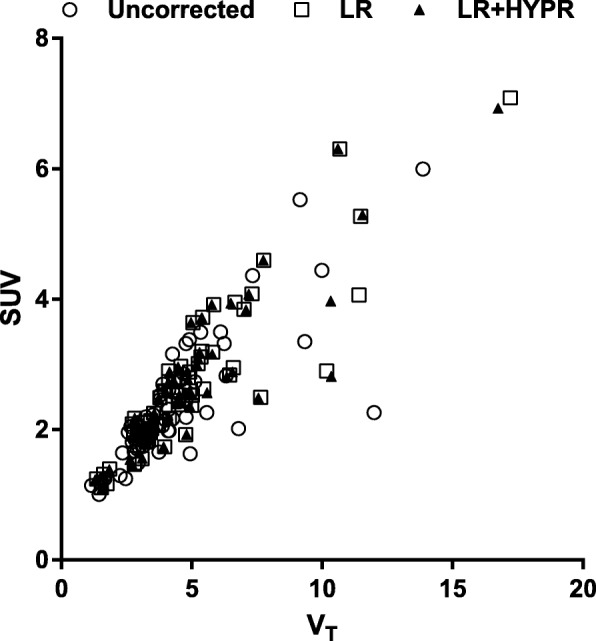
Scatter plot of *V*_T_ versus SUV, without and with PVC. For both LR and LR + HYPR, the Spearman correlation between *V*_T_ and SUV increased from 0.82 to 0.90 after PVC

PVC increased the correlations between SUV and *V*_T_ and *K*_1_, but not for BP_ND_ (Table 4). PVC increased the correlations between TBR and *V*_T_, *K*_1_, and BP_ND_ (Table 4). Largest increases in these correlations were seen between *V*_T_ and SUV (0.82 to 0.90; Fig. 4). However, confidence intervals of these correlations overlapped and therefore were not statistically significant.

**Table 4:** Correlation (Spearman, with 95% confidence intervals) between kinetic parameter estimates and simplified metrics, with and without PVC

	*V*_T_	*K*_1_	BP
Uncorrected			
SUV	0.82* (0.72–0.89)	0.43* (0.19–0.62)	0.89* (0.82–0.93)
TBR	0.81* (0.69–0.88)	0.47* (0.24–0.65)	0.82* (0.72–0.89)
LR			
SUV	0.90* (0.83–0.94)	0.45* (0.22–0.63)	0.89* (0.82–0.93)
TBR	0.88* (0.81–0.93)	0.48* (0.26–0.65)	0.84* (0.74–0.90)
LR + HYPR			
SUV	0.90* (0.83–0.94)	0.48* (0.26–0.65)	0.89* (0.81–0.93)
TBR	0.88* (0.81–0.93)	0.51* (0.30–0.68)	0.83* (0.73–0.90)

*p < 0.01

**Fig. 5:**
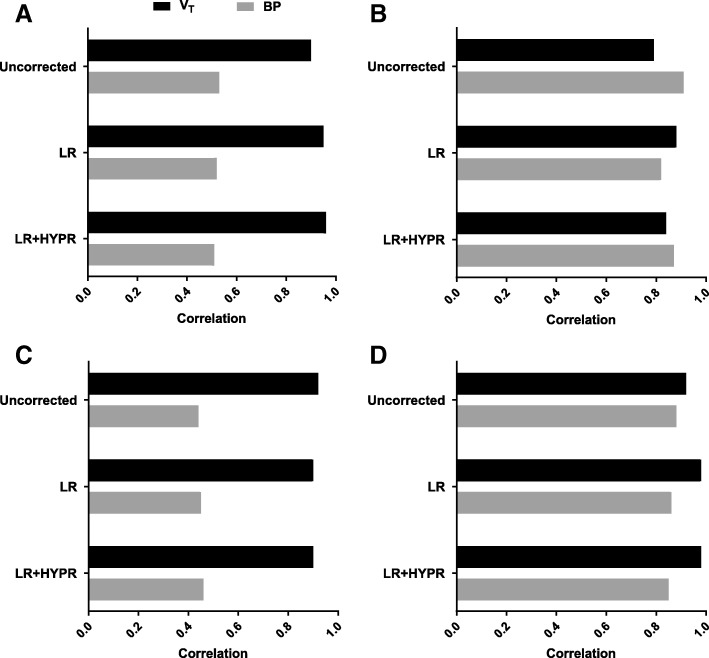
Correlation (Spearman) between changes in kinetic parameter estimates vs. simplified metrics during treatment with TKI, with and without PVC. Results shown are for SUV at 7 (**a**) and 28 (**b**) days, and for TBR at 7 (**c**) and 28 (**d**) days after treatment start

During treatment, *V*_T_, BP_ND_, SUV, and TBR significantly decreased, while *K*_1_ did not change (as was also observed in Frings et al. [5]), regardless of PVC (*p* values in Additional file 1: Table S4). At 7 and 28 days after starting treatment, original MATV demonstrated a median decrease of 16.1% (IQR − 38.9 to − 0.6), and 17.6% (IQR − 58.3 to 4.3). We correlated treatment-induced relative changes in kinetic parameters to treatment-induced relative changes in simplified metrics during treatment with TKIs for the uncorrected data as well as those with PVC (Fig. 5). At both 7 and 28 days after treatment start, changes in *V*_T_ and BP_ND_ were significantly correlated (0.79–0.98 and 0.44–0.91, respectively) with changes in SUV and TBR (with the exception of correlation between changes in BP_ND_ vs. TBR on LR images at 7 days; 0.45, *p* > 0.05), regardless of PVC. PVC (both LR and LR + HYPR) did not improve correlations between treatment induced changes in BP and changes in SUV or TBR. PVC increased the correlation between treatment-induced changes in SUV and *V*_T_ at 7 days and 28 days (increases in correlation ranging 0.05–0.09, with overlapping confidence intervals). Also, PVC increased the correlation between treatment-induced changes in TBR with changes in *V*_T_ at 28 days, but not at 7 days, after treatment start by 0.06 for both LR and LR + HYPR, with overlapping confidence intervals.

## Discussion

In the present study, we evaluated the impact of frame-wise parametric PVC on tumor kinetic parameter estimation derived from dynamic PET-CT scans and the resulting effect on validation of simplified metrics. PVC significantly increased both tumor micro- and macrokinetic parameters, and we observed that partial-volume effects varied over time due to blood pool activity and changing tumor contrast. Hence, the effect of PVC on kinetic parameter estimates was not in full concordance with its effect on simplified metrics (SUV and TBR), and as a consequence, PVC was found to affect the validation of SUV using *V*_T_ both for single measurements and as biomarker of treatment response to a small extent (albeit non-significantly).

Application of PVC in oncologic dynamic PET-CT studies is scarce. Mankoff et al. (2003) applied PVC in dynamic FDG-PET of breast cancer patients using a simple method with recovery coefficients, assuming lesions are spherical with homogenous tracer distributions [29]. They observed that applying PVC in response measurements reduced changes in metabolic rate of FDG and blood flow of responding patients, reducing significance of parameter changes (albeit still statistically significant). By using this method, however, kinetic parameters were solely corrected for (changes in) tumor size, and no correction for spill-in from blood pool structures and/or heterogeneous tumor background was applied. In 2007, Teo et al. validated the use of iterative deconvolution as an image-based PVC method not requiring anatomical segmentation or knowledge of lesion size, and suggested its potential application in kinetic modeling, which to the best of our knowledge has not been performed to date for oncologic PET-CT [30].

Both tumor macroparameters *V*_T_ and BP_ND_, and microparameter *K*_1_ significantly changed after application of PVC. This corresponds with results from applications of PVC in brain dynamic PET studies, where similar increases in kinetic parameter estimations have been observed when applying PVC in the case of activity spill-out [19–21, 31]. Interestingly, the effect of PVC on kinetic parameter estimates was poorly (albeit significantly) correlated with its effect on simplified measures. As previously described [9], the effect of PVC on SUV of (hotspot) lesions on static PET-CT scans is straightforward: an expected net increase in activity, mainly dependent on lesion size (and, in lesser extent, shape and local contrast). This can be seen in Fig. 2, where change in SUV after PVC is highly (inversely) correlated to tumor volume, while the kinetic parameter estimations are not. This illustrates that impact of PVC on tumor kinetic parameter estimation is more complex, as seen in Fig. 1 which displays the non-linear temporality of partial-volume effects for a typical mediastinal lymph node metastasis. Here, an early spill-in of activity due to blood pool proximity is noted, with increasing activity spill-out afterwards as tumor uptake increases and background activity decreases. Hence, across lesions, the effect of PVC on kinetic parameters may differ depending not only on size, but as well on the presence of proximate high activity structures, rate of tracer uptake during the scan, and background activity.

For quantification of functional tumor characteristic on PET-CT in clinical practice, a simplified quantitative method is necessary, obviating the need for complex and extended dynamic image acquisitions, need for blood sampling, and facilitating the possibility of whole-body acquisitions. To this end, per radiotracer and cancer type simplified metrics needs to be technically validated by pharmacokinetic modeling using dynamic PET-CT [4]. In the current study, the effect of PVC on kinetic parameter estimates was different from its effect on simplified metrics, which explains why it might affect validation of these simplified metrics (using *V*_T_). We observed a trend that PVC increased correspondence of SUV with *V*_T_ in single measurements (correlations improving from 0.82 to 0.90) and as a biomarker of treatment response (correlations improving from 0.90 to 0.95 at 7 days and from 0.79 to 0.88 at 28 days after treatment start). However, confidence intervals of these correlations overlapped, which might at least partly be due to the sample size (inherent to this type of study), and therefore these differences are not statistically significant. Therefore, while PVC is mandated to acquire accurate quantitative reads, it only increases correspondence of kinetic parameters with simplified metrics to a small extent on a cohort level. This indicates that the impact of image resolution on technical validation of simplified metrics of ^18^F-FLT as biomarkers of response to TKI might be small, and that PET images without PVC seem non-inferior for this purpose. It should be noted that for response assessment to treatments that affect tracer kinetics and blood pool activity to a larger extent than TKIs and for other cancer types more affected by spill-in (e.g., prostate cancer lesions with urinary tract proximity), PVC may have a larger impact on validation of simplified metrics.

Spill-out due to PVE will result in overestimation of metabolic tumor volumes, which increases the underestimation of true tracer uptake since background activity is included [11]. A parametric PVC method may therefore theoretically reduce inaccuracies in delineation. However, iterative deconvolution has been proposed with use of VOIs defined on uncorrected images, due to the expected propagation of image noise after PVC [30]. We evaluated the impact of delineation on deconvoluted images with HYPR denoising, and found not only substantial decreases in MATVs (Fig. 3) but also an increase in PVCs effect on kinetic parameter estimates (Additional file 1: Table S3). Nonetheless, our previous study demonstrated that the reduction in MATV after PVC may not necessarily lead to more accurate definition of tumor volumes [11].

In brain PET studies, frequently a small vessel such as the carotid artery needs to be utilized for IDIF generation. This mandates PVC due to the small artery diameter [32, 33]. In this study on thoracic oncological PET-CTs, the ascending aorta, a large vessel, was used for IDIF generation. We noted that PVC introduced negligible differences in IDIF area under the curves, and that without denoising this introduced small but significant differences in kinetic parameter estimates (Additional file 1: Table S2). However, since HYPR denoising using a single composite image (providing maximum noise reduction) appeared to completely mitigate this effect, the effect of PVC on these input functions seems to be based on PVC-induced noise-propagation. Therefore, when input functions derived from large blood pool structures are used, PVC is preferably avoided to evade noise-induced inaccuracies in kinetic parameter estimates (assuming no spillover from nearby high activity structures).

Iterative deconvolution algorithms are known to propagate image noise, which may necessitate denoising methods to be applied to preserve image quality. Several approaches have been proposed, such as wavelet-based denoising for static PET-CT and HYPR denoising for dynamic acquisitions, respectively [26, 34]. We observed that HYPR needs to be optimized for tracer kinetics using a moving composite image, since when applied using a single composite image (maximal denoising) it seems to lose the temporal dynamic course of the PVC (Fig. 1). Including HYPR_moving_ resulted in very similar outcomes compared to PVC alone, and slightly mitigated the increase in kinetic parameter estimates after PVC. The latter may not only be attributed to reduced statistical noise but also to some smoothing effects inherent to the algorithm. Also, at late time frames, it had no effect on intratumoral COV% (Additional file 1: Figure S1). This might be explained by the high tumor contrast and high count number (due to the long frame duration), as Golla et al. previously demonstrated [21]. The increase in COV% at late time frames thus seems to be a resultant of increased intratumoral heterogeneity by PVC itself. Therefore, in region-based non-linear regression analyses, the impact of PVC-induced increased image noise on kinetic parameter estimation seems negligible. However, it may have significant impact when tumors are analyzed on a parametric level.

While the presence of PVE and the consequent need for PVC are well recognized, to date PVC has rarely been applied in oncological PET studies. This may be because to date there is no consensus on the optimal correction strategy and data yielded from application of PVC does not seem to have triggered routine clinical application [12, 35]. Our study now demonstrates that PVC should not only be performed in future regular static PET-CT studies, but in dynamic PET-CT studies as well, also when simplified quantitative metrics are validated for clinical applications. If not applied, small lesions should preferably be excluded from analyses, as recommended and performed in previous studies using a 2–3-cm-diameter cut-off to avoid PVE [36, 37]. Still, our data demonstrate that lesions above these size thresholds are also affected by PVE (Fig. 2).

Only data from ^18^F-FLT PET-CT was used. However, the current dataset from a widely used whole body TOF PET-CT scanner allowed for both kinetic modeling and extraction of simplified parameters per lesion, at time points used in clinical practice due to the long acquisition time (0–60 min post-injection). Also, the dataset included both large and small lesions, both nearby and remote from large blood pool structures. Additionally, it facilitated evaluation of PVCs effect on validation of simplified parameters both in single measurements and during systemic treatment. Since we have demonstrated the significant effect of PVC in kinetic parameter estimation, future dynamic PET studies focusing on other PET-tracers in small tumors (e.g., PSMA-ligand tracers in prostate cancer metastases) should apply PVC as a similar (or larger) impact of PVC may be expected. In the current study, no correction was made for potential motion blurring effects, which is another factor possibly affecting accuracy of kinetic parameter estimations [38]. Efforts should be made to incorporate both PVC and motion correction methodologies simultaneously for dynamic PET studies. Also, the impact of PVC on parametric kinetic analyses of oncologic dynamic PET warrants further investigation, which will require HYPR denoising to be optimized for this purpose.

## Conclusion

Parametric PVC using iterative deconvolution had a significant impact on tumor kinetic macro- and microparameter estimations from dynamic PET-CT. The relative effects of PVC on kinetic parameter estimations and simplified metrics were poorly correlated. This resulted in a non-significant trend in higher correlation between *V*_T_ and SUV in single reads and affected its technical validation as a biomarker of treatment response to a small extent. Therefore, the impact of image resolution on technical validation of simplified metrics for clinical use seems to be small. When optimized according to tracer kinetics, HYPR denoising may adequately reduce PVC-induced image noise for low count and low contrast timeframes. However, it has only limited effect on kinetic parameter estimations and thus may be obviated for region-based non-linear regression analysis. Future oncologic dynamic PET-CT studies should preferably incorporate PVC to acquire accurate quantitative reads.

## Additional file

Additional file 1: **Table S1.** Spearman correlations between IDIF AUCs of PVC-images and uncorrected images. All correlations were significant with *p* < 0.001. **Table S2.** Median relative differences (% with IQR) in K1, Vt, and k3/k4 of uncorrected images using uncorrected versus corrected IDIFs (PVC without and with HYPR denoising). *p < 0.05 Wilcoxon-signed-rank test. **Table S3. **Relative changes (%) in kinetic parameter estimates and simplified metrics after PVC using VOIs delineated on PVC images (LR + HYPR). **Table S4.** *P*-values of testing (Friedman’s test) between changes in kinetic parameter estimates and simplified metrics (with and without PVC) during treatment with TKI at 7 and 28 days after treatment start. **Figure S1.** Time-activity curves of intralesional image noise (COV%) without and with PVC using several HYPR settings. Frames of 0–4 min (A) and 4–60 min (B) post-injection. Results of a typical mediastinal lymph node metastasis are shown. (DOCX 450 kb)

## Abbreviations

^18^F-FLT: 3′-Deoxy-3′-^18^F-fluorothymidine
AUC: Area under the curve
BP_ND_: Non-displaceable binding potential
HYPR: Highly constrained backprojection
IDIF: Image-derived input function
*K*_1_: Influx of tracer from blood to tissue
LR: Lucy-Richardson iterative deconvolution
NSCLC: Non-small cell lung cancer
PET: Positron-emission tomography
PVC: Partial-volume correction
SUV: Standardized uptake value
TBR: Tumor-to-blood ratio
TKI: Tyrosine kinase inhibitor
VOI: Volume of interest
V_T_: Volume of distribution
