# Supplementary material for: Partial-volume correction in dynamic PET-CT: effect on tumor kinetic parameter estimation and validation of simplified metrics
Source: EJNMMI Res. 2019 Feb 4;9:12. doi: 10.1186/s13550-019-0483-z (PMC6362178; doi:10.1186/s13550-019-0483-z)

**Table S1:** Spearman correlations between IDIF AUCs of PVC-images and uncorrected images. All correlations were significant with p<0.001.

|  | Entire curve | | Peak only (2.5 min) | |
| --- | --- | --- | --- | --- |
|  | **Image-derived** | **Calibrated** | **Image-derived** | **Calibrated** |
| LR | 0.994 | 0.991 | 0.993 | 0.988 |
| LR+HYPR (-/+ 3 frames) | 0.994 | 0.990 | 0.992 | 0.987 |
| LR+HYPR (single composite) | 0.994 | 0.992 | 0.997 | 0.996 |

**Table S2:** Median relative differences (% with IQR) in K1, Vt, and k3/k4 of uncorrected images using uncorrected versus corrected IDIFs (PVC without and with HYPR denoising). *p<0.05 Wilcoxon-signed-rank test.

| IDIF: | K1 | Vt | k3/k4 |
| --- | --- | --- | --- |
| LR | 2.9 (0.2 to 7.4)* | 0.9 (-1.1 to 2.6)* | -0.2 (-3.7 to 2.4) |
| LR+HYPR (-/+ 3 frames) | 3.1 (0.9 to 5.5)* | 1.1 (-0.4 to 4.5)* | -0.8 (-3.2 to 1.6) |
| LR+HYPR (single composite) | 0.0 (-0.2 to 0.1) | 0.0 (0.0 to 0.1) | 0.1 (-0.1 to 0.1) |

**Table S3:** Relative changes (%) in kinetic parameter estimates and simplified metrics after PVC using VOIs delineated on PVC images (LR+HYPR).

|  | Mean | Median | SD | IQR | Min | Max | p-value |
| --- | --- | --- | --- | --- | --- | --- | --- |
| LR+HYPR: | | | | | | | |
| Vt | 13.6 | 13.9 | 7.9 | 7.6 – 18.7 | 5.5 | 37.8 | <0.001 |
| K1 | 6.6 | 5.3 | 6.7 | 2.5 – 10.1 | 4.4 | 30.3 | <0.001 |
| BP | 5.7 | 5.4 | 7.6 | 1.2 – 8.9 | 3.7 | 38 | <0.001 |
| SUV | 15.4 | 15.8 | 7.1 | 8.4 – 20.4 | 3.6 | 31.5 | <0.001 |
| TBR | 15.6 | 15.8 | 7.2 | 8.4 – 20.7 | 5.7 | 34.0 | <0.001 |

**Table S4**: P-values of testing (Friedman’s test) between changes in kinetic parameter estimates and simplified metrics (with and without PVC) during treatment with TKI at 7 and 28 days after treatment start.

|  | Vt | K1 | BP | SUV | TBR |
| --- | --- | --- | --- | --- | --- |
| Uncorrected | <0.001 | 0.45 | 0.038 | 0.001 | 0.002 |
| LR | <0.001 | 0.819 | 0.005 | 0.001 | 0.002 |
| LR+HYPR | <0.001 | 0.819 | 0.031 | 0.001 | 0.002 |

**Figure S1: Time-activity curves of intralesional image noise (COV%) without and with PVC using several HYPR settings.** Frames of 0-4 minutes (A) and 4-60 minutes (B) post-injection. Results of a typical mediastinal lymph node metastasis are shown.


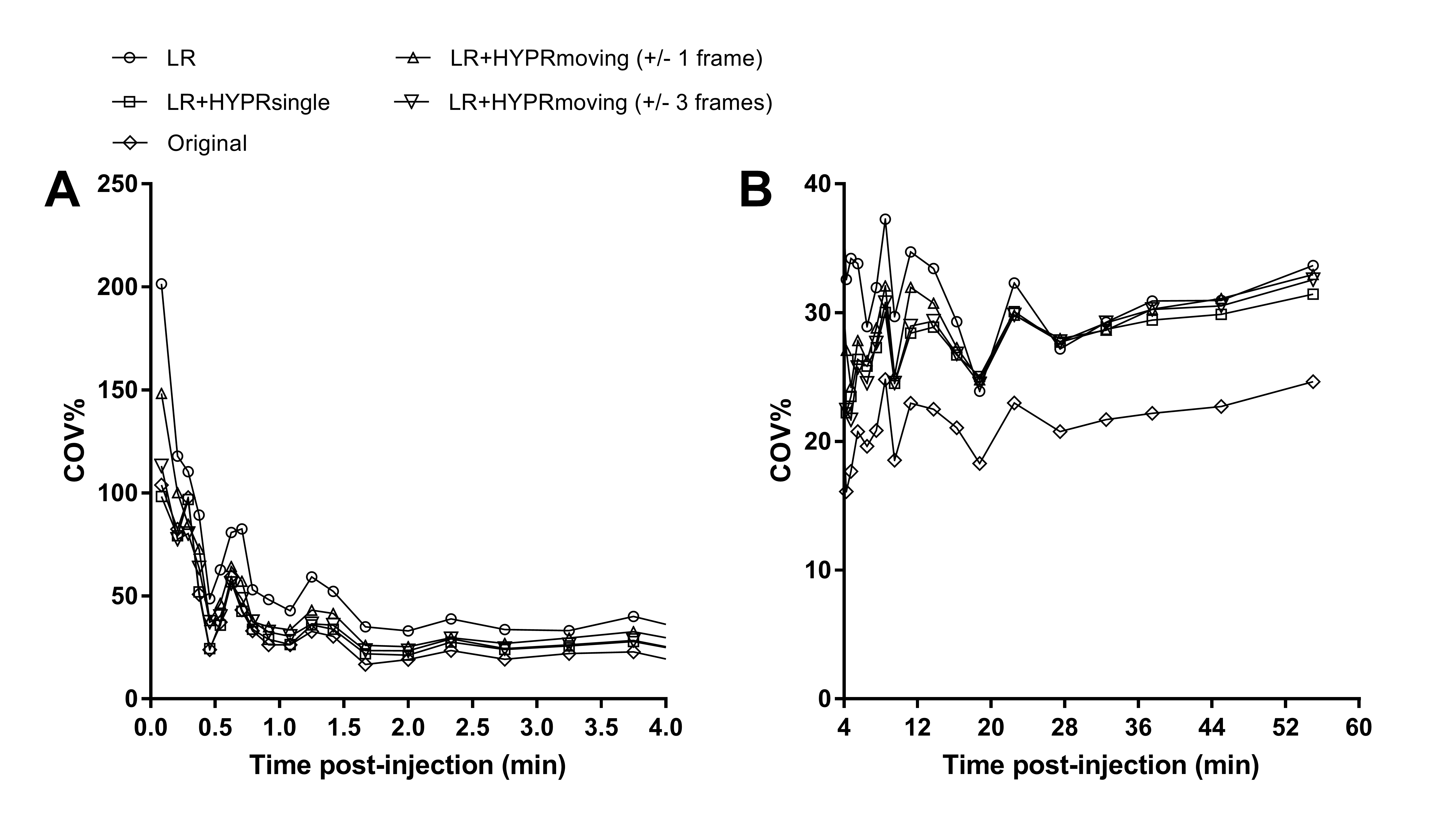

Supplement: Supplementary file 1 — Table S1. Spearman correlations between IDIF AUCs of PVC-images and uncorrected images. All correlations were significant with p < 0.001. Table S2. Median relative differences (% with IQR) in K1, Vt, and k3/k4 of uncorrected images using uncorrected versus corrected IDIFs (PVC without and with HYPR denoising). *p < 0.05 Wilcoxon-signed-rank test. Table S3. Relative changes (%) in kinetic parameter estimates and simplified metrics after PVC using VOIs delineated on PVC images (LR + HYPR). Table S4. P-values of testing (Friedman’s test) between changes in kinetic parameter estimates and simplified metrics (with and without PVC) during treatment with TKI at 7 and 28 days after treatment start. Figure S1. Time-activity curves of intralesional image noise (COV%) without and with PVC using several HYPR settings. Frames of 0–4 min (A) and 4–60 min (B) post-injection. Results of a typical mediastinal lymph node metastasis are shown. (DOCX 450 kb) [file 13550_2019_483_MOESM1_ESM.docx]
